# Drivers of solar radiation variability in the McMurdo Dry Valleys, Antarctica

**DOI:** 10.1038/s41598-018-23390-7

**Published:** 2018-03-22

**Authors:** M. K. Obryk, A. G. Fountain, P. T. Doran, W. B. Lyons, R. Eastman

**Affiliations:** 1 U.S. Geological Survey, Cascades Volcano Observatory, Vancouver, 98683 WA USA; 2 Portland State University, Department of Geology, Portland, 97201 OR USA; 3 Louisiana State University, Department of Geology and Geophysics, Baton Rouge, 70803 LA USA; 4 The Ohio State University, Byrd Polar Research Center, Columbus, 43210 OH USA; 5 University of Washington, Department of Atmospheric Sciences, Seattle, 98195 WA USA

## Abstract

Annually averaged solar radiation in the McMurdo Dry Valleys, Antarctica has varied by over 20 W m^−2^ during the past three decades; however, the drivers of this variability are unknown. Because small differences in radiation are important to water availability and ecosystem functioning in polar deserts, determining the causes are important to predictions of future desert processes. We examine the potential drivers of solar variability and systematically eliminate all but stratospheric sulfur dioxide. We argue that increases in stratospheric sulfur dioxide increase stratospheric aerosol optical depth and decrease solar intensity. Because of the polar location of the McMurdo Dry Valleys (77–78°S) and relatively long solar ray path through the stratosphere, terrestrial solar intensity is sensitive to small differences in stratospheric transmissivity. Important sources of sulfur dioxide include natural (wildfires and volcanic eruptions) and anthropogenic emission.

## Introduction

Variations in solar radiation reaching the top of the atmosphere that result from rotational and orbital cycles of the Earth as well as variations of the sunspot cycle are well-known. However, the intensity of solar radiation actually reaching the Earth’s surface is proportional to atmospheric optical depth^1^, which includes path length through the atmosphere and turbidity (e.g. water vapor, cloud cover, and other aerosols). The optical depth is modulated by terrestrial processes including volcanic eruptions^2^, wildfires^3^, and human-induced air pollution^1^, which increase aerosol concentrations and form cloud condensation nuclei. Sometimes these effects can have profound impacts on climate. For example, the Little Ice Age may have been initiated by heightened volcanic activity, during which large volumes of aerosols were injected into the stratosphere, reducing solar radiation and triggering a feedback that cooled the Northern Hemisphere for 500 years^4^. Sulfate aerosols in the atmosphere (troposphere and stratosphere) can affect terrestrial solar intensity at the surface by scattering solar radiation and by forming cloud condensation nuclei; both processes have negative forcings on the Earth’s radiative balance^5–8^. Recently, numerous long-term solar radiation studies in Europe and Asia showed decreasing solar radiation trends until the 1990s, followed by an increase thereafter^5,9^. These variations have been attributed to variable aerosol concentrations, primarily sulfates^1,5,8–10^.

**Figure 1:**
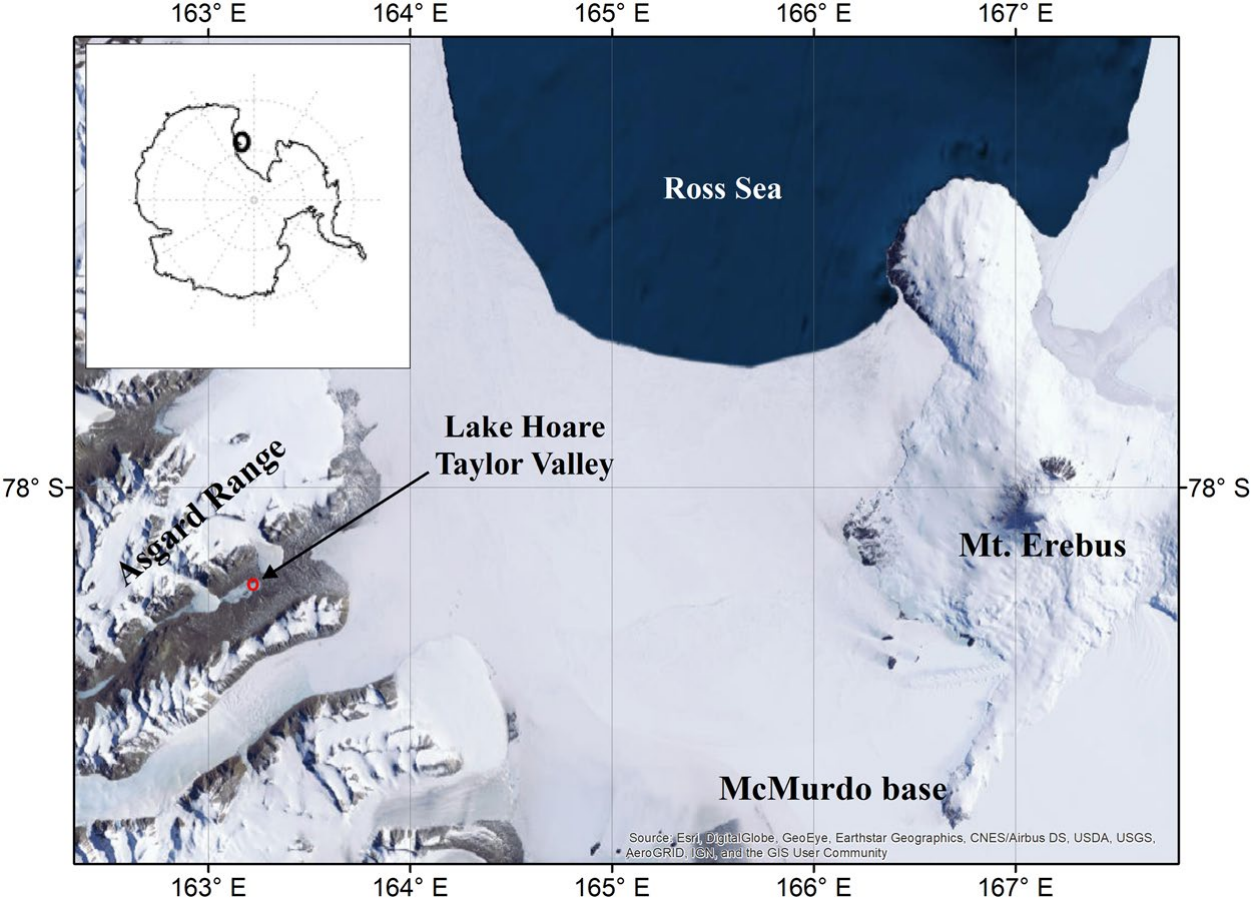
Map of McMurdo Dry Valleys, Antarctica. Lake Hoare station is located in Taylor Valley. Map generated in ArcGIS 10.1; Antarctica insert generated in Matlab.

Here we explore the causes of variations in annual solar radiation intensity in the McMurdo Dry Valleys, Antarctica (MDV; 77–78°S 160–164°E) (Fig. 1), a pristine environment, devoid of direct industrial pollution^11,12^. The MDV is the largest ice-free area on the Antarctic continent^13^, and lies within the Transantarctic Mountains with peaks of about 2000 m and valley floors between 10 s to 100 s meters above sea level. Mean annual air temperature on the valley floor ranges from −14.8 °C to −30.0 °C^14^ and annual precipitation is <50 mm water equivalent^15^. During the 1990s, summer air temperatures were cooling at a rate of 0.7 °C decade^−1^ and solar radiation was increasing at a rate of about +8 W m^−2^ decade^−1^; since 2000 no trends are apparent^12,14^. The hydrology of the region, dominated by glacial melt water, is very sensitive to small variations in energy balance, and solar intensity is quite important for generating glacial melt and controlling soil temperatures, including the active layer depth (maximum seasonal soil thaw depth)^11,16^. Small change of the surface energy balance, as a consequence of atmospheric opacity, can have profound effects on melt water potential during austral summers^16^. The valleys also include a microbially-dominated ecosystem whose functioning is controlled by available energy^16,17^.

## Results and Discussion

**Figure 2:**
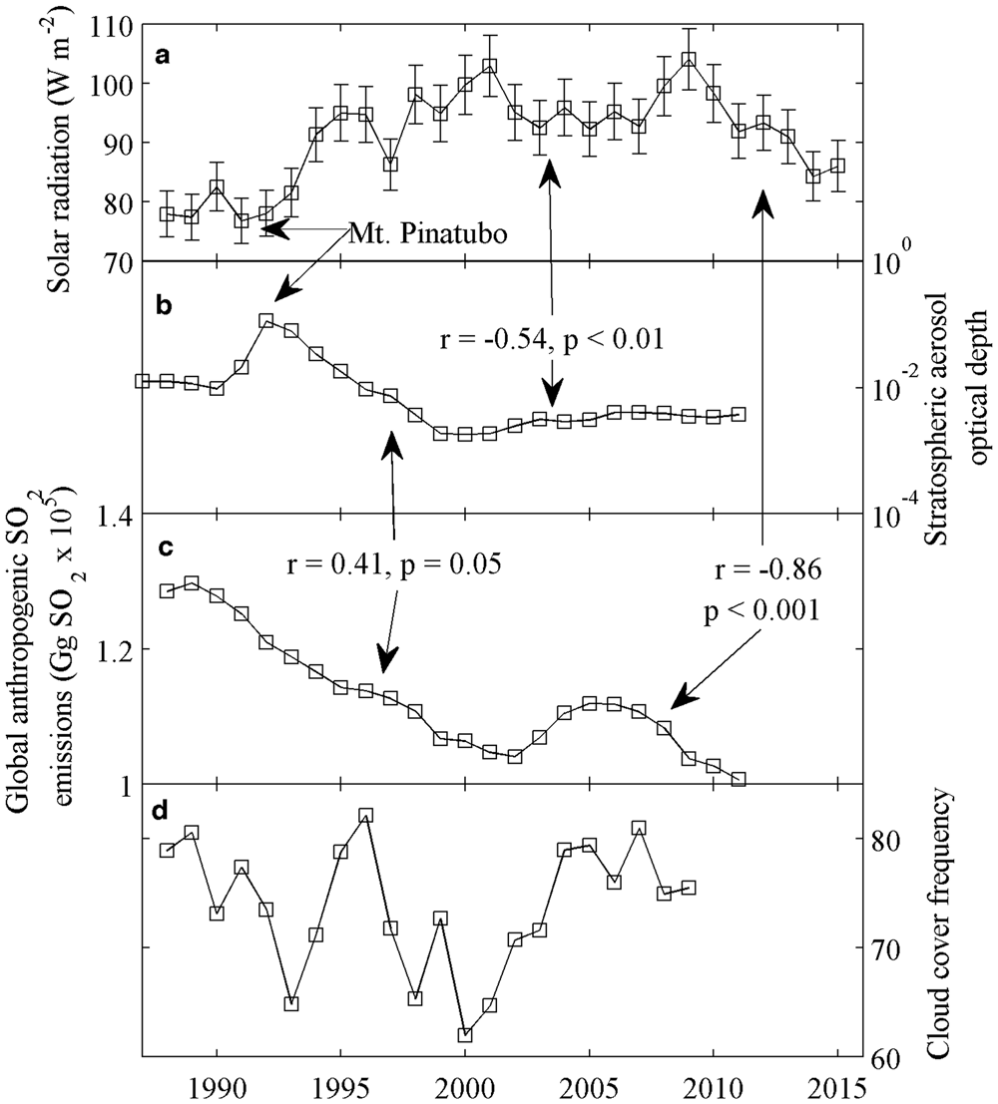
Variations in solar radiation measured in the McMurdo Dry Valleys compared to possible atmospheric forcings. (**A**) Annually averaged measured solar radiation at Lake Hoare, McMurdo Dry Valleys meteorological station. Error bars are shown as maximum possible sensor error. (**B**) Stratospheric aerosol optical depth at 550 nm between 15 and 20 km altitude and latitude between 74°S to 82°S. (**C**) Annually averaged global anthropogenic SO_2_ emissions. (**D**) Annually averaged middle and high cloud cover data at McMurdo Dry Valleys (~100 km away from Lake Hoare meteorological station).

Annual mean values of shortwave radiation increased by about 25 W m^−2^ from the start of measurements in 1987 (78 W m^−2^) to a peak in 2001 (103 W m^−2^) (see methods for uncertainty). Between 2001 and 2009, the radiation decreased to a minimum of 92 W m^−2^ in 2003 then increased again through 2009. Since 2009, the radiation has decreased by 18 W m^−2^ to 86 W m^−2^ in 2015 (Fig. 2a and Supplementary Figure S1 for fitted long-term moving average). These changes are quite large but we believe them to be real because the same pattern was observed at seven other independent solar radiation stations in the valley (Supplementary Figure S2) and the sensors are recalibrated every two years (see methods).

We first considered changes in cloud cover and Southern Annular Mode (SAM) magnitude as drivers of solar radiation variability. No temporal cloud cover data exists for the MDV, so we utilized cloud cover data from the nearby McMurdo Station located on the Ross Island^18^, approximately 100 km away. To accurately represent clouds in the McMurdo Dry Valleys, we excluded low clouds (e.g. nimbostratus) as they may create overcast conditions local to Ross Island but are likely not present in the MDV (personal communication, R. Eastman). Seasonally averaged (October to April) non-precipitating middle and high clouds, expressed as frequency of occurrence, are negatively correlated to solar radiation but are not statistically significant (Table 1). Although cloud cover can be approximated utilizing measured solar radiation to the clear-sky solar radiation ratio, these estimates are contaminated by the very scattering processes that we wish to investigate, rendering the estimates useless.

**Table 1:** Correlation coefficients between solar radiation (W m^−2^) at Lake Hoare, Taylor Valley, Antarctica and stratospheric aerosol optical depth (SAOD, m), Southern Annual Mode (SAM), maximum sea ice extent (MSIE, km^2^), ‘Snow pit’ sulfate aerosols (μg L), ‘Clouds’ mid- to high-cloud expressed as frequency of occurrence over the McMurdo Station, and global anthropogenic sulfate aerosols (Gg SO_2_). Statistically significant relations are in bold. In parenthesis, statistics without the influence of volcanic or wildfire events.

	Solar radiation	SAM	MSIE	Snow pits SO_4_	Clouds	SO_2_
Solar radiation		r = 0.27 p = 0.16	r = 0.37 p = 0.06	r = −0.28 p = 0.36	r = −0.25 p = 0.25	**r = −0**.**86** (**r = −0**.**94**) **p < 0**.**001** (**p < 0**.**001**)
SAOD	**r = −0**.**54 p = 0**.**006**	r = −0.09 p = 0.66	**r = −0**.**48 p = 0**.**01**	r = 0.47 p = 0.11	r = −0.15 p = 0.51	**r = 0**.**41 p = 0**.**05**

The Southern Annular Mode (SAM), which is the first empirical orthogonal function of the pressure field over Antarctica, controls the strength of the prevailing winds (westerlies) around Antarctica and is the main driver of atmospheric variability in the Southern Hemisphere^19–21^. As such, the predominantly zonal westerly winds around Antarctica can affect tropospheric aerosol exchange with the lower latitudes and may affect local atmospheric transmissivity. During the positive SAM, the zonal winds move toward Antarctica, allowing tropospheric aerosol transport closer to the Antarctic continent^22^. Conversely, during the negative SAM, the zonal wind belt moves toward the equator, insulating higher latitudes^22^. We found no significant correlation between annually and seasonally averaged SAM index and MDV solar radiation (Table 1).

Possible effects of the ozone hole variability were dismissed despite the increased ultraviolet radiation intensity that occurs^23^. Our sensors only measure wavelengths of 400 to 1100 nm (see methods) and are insensitive to increased irradiance in ultraviolet wavelengths.

Regionally and globally, short-term temporal variations of incident solar radiation have been attributed to variations in sulfate aerosols, which can form from sulfur dioxide in the atmosphere^1,5,8–10^. Sulfur dioxide is the precursor to sulfate aerosol formation^8,10^, and is emitted from natural sources (e.g. wildfires, dimethylsulfide from marine phytoplankton, volcanic eruptions) and anthropogenic sources (fossil fuel burning)^24^. The majority of sulfate aerosols in the atmosphere are of anthropogenic origin, mostly from industrialized regions^6,8,9,25^.

Regional sources, local to the MDV, of sulfur dioxide may include dimethylsulfide (DMS) from the Ross Sea/McMurdo Sound, volcanic emissions from Mount Erebus, and anthropogenic pollution from McMurdo Station and Scott Base. DMS is a biogenically-derived precursor to the sulfate aerosol formation (e.g. methanesulphonate or non-sea-salt-SO_4_^26^). In 1980’s, DMS has been hypothesized to increase cloud condensation nuclei in the marine boundary layer and a consequent reduction of solar radiation due to the increased cloud cover formation^26^. However, recent studies of biogenic control of cloud condensation nuclei in remote marine boundary layer have questioned this hypothesis^27^ and showed that the variable DMS emission rates have very limited effect on the cloud condensation nuclei formation^28^. Nonetheless, we examine the DMS hypothesis for our site. In polar regions, DMS production results from seasonal phytoplankton blooms due to variations in sea ice extent^29,30^. A larger sea ice extent, and its subsequent breakup, encourages greater phytoplankton blooms and elevated DMS fluxes to the atmosphere^31^. Time series of biogenically-derived sulfur gases retrieved from ice cores at Law Dome^29^ and from snow pits in glaciers located near the McMurdo Dry Valleys^32^ were positively correlated with sea ice extent. Therefore, a larger sea ice extent in the adjacent Ross Sea should be associated with greater DMS production, higher concentrations of sulfate aerosols, and lower solar radiation (a negative relationship between sea ice extent and solar radiation). However, the results show positive correlation (r = 0.37, p = 0.06, n = 26) between maximum sea ice extent in the Ross Sea region and solar radiation (Table 1). There are five Antarctic sea ice sectors determined based on similarity of their spatial behavior^33^ and the Ross Sea ice sector encompasses an area between longitudes 160°E–130°W^33,34^. Comparisons between non-sea-salt-SO_4_ concentrations obtained from the snow pits around the MDV (unpublished data B. Lyons) and solar radiation show a lack of statistical significance (Table 1). Collectively, these results suggest that regional DMS release does not affect local solar intensity at our study site, conforming with previously published work on DMS and cloud condensation nuclei formation^27,28^.

Mount Erebus, a 3794 m tall, open vent, and continuously degassing volcano^35^ is located on Ross Island about 100 km to the southeast of our meteorological station at Lake Hoare (Fig. 1). Sulfur dioxide is not continuously monitored and emission rates are measured only during the month of December^35^. Averaged annual SO_2_ emission rates vary between 52 to 74 t d^−1^ and increased over the period 1984 to ~1992 after which they remained relatively constant until 2006^35,36^. Using satellite-derived annual SO_2_ emission rates for Mount Erebus, available for 2005 and 2016 (55% uncertainty)^37^, show a statistically significant correlation with observed solar radiation at Lake Hoare over the last decade (r = 0.60, p = 0.05, n = 11). The increase of sulfur dioxide emission is inconsistent with an increase of solar radiation over the same time period.

Local anthropogenic sources (e.g. diesel generators, terrestrial and aerial traffic, excluding ships) are two orders of magnitude lower than the Mount Erebus contribution^36^. Unlike other coastal regions around Antarctica, few ships visit the Ross Sea making their emissions of sulfur dioxide a minor contribution^36^. Consequently, local anthropogenic sources of sulfate aerosols are an unlikely candidate of solar radiation variability in the MDV. Note, by directly comparing local emissions (natural and anthropogenic) with solar radiation values at our meteorological station, we assumed that all local aerosol sources contribute to solar radiation variability. This assumption eliminates the need for local wind analysis; the wind component would dilute aerosol concentrations and weaken any relationship.

### Global aerosol sources

For sources of sulfate aerosols outside of Antarctica, atmospheric optical depth, transport mechanisms and aerosol residence time become important factors. In the troposphere, the residence time of sulfate aerosols is short (~weeks) due to oxidation processes and a combination of wet and dry deposition; consequently transport distances are relatively short and limited to within the hemisphere of origin^38,39^. In contrast, residence time in the stratosphere is on the order of years, allowing for inter-hemispherical mixing and global distribution^40^. The removal of stratospheric aerosols is mostly due to gravitational settling because stratospheric temperature inversion inhibits vertical mixing, permitting aerosol migration toward the poles via Brewer-Dobson circulation^41^. However, transport of aerosols to the stratosphere is typically capped by the tropopause. Under certain conditions, the natural convection of warm, humid air parcels causes them to overshoot the tropopause and enter the stratosphere^42^. This upwelling is largely limited to the equatorial regions where heat and humidity are sufficiently large to create the necessary conditions. The second process for aerosol injection to the stratosphere is volcanic eruptions whereby the explosive power of the eruption can send the gas and fine particulates directly into the stratosphere^2,43^.

We contend that polar regions may be particularly sensitive to relatively low concentrations of aerosols in the stratosphere. The length of the ray path of sunlight to the MDV is greater than nadir by a factor of 1.5 to ~14, depending on the time of year, time of day, and assuming a stratosphere height of 50 km. Estimates of stratospheric sulfate aerosols were obtained from satellite measurements of stratospheric aerosol optical depth (SAOD), defined as an integrated attenuation (extinction coefficient at 550 nm) of solar radiation at nadir. However, 30% to 70% of aerosols contributing to the SAOD are below satellite detection height (the bottom of the stratosphere in high latitudes is several kilometers lower)^44^. For this reason, SAOD in high latitudes may not accurately reflect the aerosol concentration.

Overall, the annually averaged SAOD (between latitudes 74°S to 82°S and altitude range between 15 to 20 km) is negatively correlated with annually averaged solar radiation at Lake Hoare station (r = −0.54, p < 0.01; Fig. 2b, Table 1). The two largest natural sulfur dioxide contributors to the atmosphere are volcanic activity and wildfires. Two relatively recent and particularly large sources of sulfur dioxide are the volcanic eruptions of El Chichón in 1982 and Mount Pinatubo in 1991, both located in low latitudes. These eruptions injected large volumes (~7 and 20 Mt, respectively) of sulfur dioxide directly into the stratosphere, globally decreasing solar radiation received at the Earth’s surface for several years afterward^2,43,45^. Our solar radiation data begin in 1987, five years after El Chichón, but prior to the Mount Pinatubo eruption. Significant decrease in solar radiation, starting in 1991 coincides with a sharp increase of SAOD, implicating the Mount Pinatubo eruption as a source (Fig. 2b and Supplementary Figure S2). This large stratospheric perturbation masks small SAOD variability prior and post the eruption (for detailed analysis of the SAOD post Mount Pinatubo eruption we refer the readers to the literature^42,44,46–48^). While Mount Pinatubo decreased solar radiation by ~10 W m^−2^ proximal to its location^42^, we record a similar decrease at our remote site, the magnitude of which we attribute to the long optical path of the sunray through the atmosphere. Since Mount Pinatubo, volcanic eruptions have occurred in both hemispheres but with much smaller sulfur dioxide emissions^49,50^. Ridley, *et al*.^44^ showed that small volcanic eruptions can influence low- to mid-latitude SOAD but it is unclear whether they had any high latitude effects. The other, smaller, solar radiation minima observed in Fig. 2a (and Supplementary Figure S3) are more difficult to explain.

Other possible sources of sulfate aerosols include wildfires. The observed decrease in solar radiation in 1997 is coincident with a period of large wildfires in the Asian-Pacific region (located between 20°N and 20°S)^3^. The net radiative forcings of the 1997 Asian wildfires were as high as −150 W m^−2^ directly over the wildfires and up to −10 W m^−2^ over the Indian Ocean^3^. Streets, *et al*.^9^ showed an increased atmospheric optical depth over Southeast Asia between 1991 and 1997 and attributed it to both Mount Pinatubo and wildfires. We speculate that the 1997 solar radiation decline in the MDV (Fig. 2a and Supplementary Figure S3) is a result of these Asian wildfires forcing convection into the stratosphere^39^. However, the decreases in MDV solar radiation in 1991 and 1997 are of similar magnitude, whereas the SAOD responded only to the Mount Pinatubo eruption (Fig. 2b and Supplementary Figure S3). This discrepancy may result from the detection bias in the satellite SAOD measurements, which are restricted to the tropopause (~15 km) and higher, and do not account for the lowered tropopause in the high latitudes (~8 km)^44^ and associated aerosols.

In the early 2000s, globally variable but increasing concentrations of stratospheric sulfate aerosols have been attributed to anthropogenic sources^47,48^ and/or small volcanic eruptions^42,44,46^. To test whether anthropogenic sources of sulfate aerosols may be important to solar intensity in the MDV, we use the annually averaged emission flux of global anthropogenic sulfur dioxide emissions^51,52^. A global inventory of anthropogenic sulfur dioxide emissions for each country was calculated based on emission reports and modeling estimates of combustion from coal, petroleum, biomass, natural gas, agricultural waste burning, metal smelting, and paper processing^51,52^. Comparing annual anthropogenic sulfur dioxide emissions to solar radiation in the MDV shows a statistically significant negative correlation (r = −0.86, p < 0.001, n = 24) (Table 1; Fig. 2c), suggesting that global anthropogenic sulfur emissions also affect solar radiation trends in the MDV. If the higher frequency events caused by volcanic eruptions and wildfires are removed by applying a long-term moving average, the correlation increases significantly (r = −0.94, p < 0.001, n = 24: Supplementary Figure S1).

The long-term solar radiation trend in the MDV resembles other studies on global dimming and brightening. Across the globe, prominent decrease in solar radiation was observed until late 1980s with a transition to global brightening in 1990s^5^. This transition was attributed to economic development and changing environmental policies^5^. A similar transition is observed in Fig. 2, ignoring the effect of Mount Pinatubo. Prior to 1991, background levels of SAOD are relatively higher than post 1999. Similarly, average solar radiation in 1980s was significantly lower than post 1999. These global trends have been attributed to changes in anthropogenic emissions, mainly sulfates^1,5,8–10^. Here we show that our Antarctic site is not an exception to this trend and the solar radiation is driven by global emissions (Supplementary Figure S1).

## Conclusions

Our analysis suggests that global sources of sulfur dioxide, which evolve to sulfate aerosols drive large variations in the intensity of solar radiation in the MDV. The sources are large volcanic eruptions, large tropical wildfires, and global anthropogenic emissions. We argue that the solar intensity in polar regions in general and specifically in the MDV is sensitive to small variations in optical depth due to the long ray path of solar radiation through the stratosphere.

We examined the potential roles of local cloud cover, proxies for hemispheric wind intensity, and local sources of sulfur dioxide, but found no significant correlations or physical basis for an influence. We recognize our reliance on limited available datasets and for this reason, we consider our conclusion a working hypothesis that will motivate future studies. If future studies support our conclusion then variations of solar radiation in the MDV are a global proxy for global sulfur dioxide emissions.

## Methods

Solar radiation is well measured in the MDV. Eight meteorological stations record solar radiation using LiCOR radiometers^53^. The correlation between all stations is statistically significant at p < 0.05 (Supplementary Figure S2 and Table S1). The LiCOR sensors used are the LiCor LI200S or LI200X silicon pyranometers (for metadata see www.mcmlter.org). These sensors are sensitive to wavelengths from 400 to 1100 nm with a maximum error of ±5%; they were factory recalibrated every two years. Data are recorded at 15-min intervals on Campbell Scientific CR10 and CR10x data loggers.

The radiation sensor at Lake Hoare, MDV (Fig. 1) was used for this study because it has the longest temporal record in the MDV (1987 to present). The meteorological station is located 77.1 m above mean sea level and 15 km inland from the coast. The radiation sensor is located three meters above ground and receives direct and diffuse radiation for about 7 months a year. Solar radiation from late August to late April represents effectively 100% of the total annual radiation received at this station. It is shadowed daily due to the adjacent mountains and large solar zenith angle variability^17^. Daily variability of solar radiation is the lowest during the summer solstice when zenith angles are the highest (54.5° to 78.5°)^17^. However, even in December, Lake Hoare station is shaded up to 3 hours daily^17^.

Solar radiation data were obtained from www.mcmlter.org. Gaps in the data occurred. Recently, a spatial meteorological model was developed for the Taylor Valley, based on eight meteorological stations within the valley (including Lake Hoare), allowing the gaps from missing data to be filled^16^. We used the model data for accurate annual averaging. Sea ice extent data were obtained from National Snow and Ice Data Center (http://nsidc.org). Southern Annual Mode data were obtained from http://www.nerc-bas.ac.uk. Sulfur dioxide data from Mount Erebus between 1984 and 2006 were collected using correlation spectroscopy and ultraviolet spectrometers with the maximum total error of ±8%^35^. Annually averaged stratospheric optical depth data obtained from data.giss.nasa.gov/modelforce/strataer/^54^. Annually averaged SO_2_ emissions data obtained from^51,52^.

The statistical significance of correlation coefficients presented in this paper does not account for the autocorrelation, which if present in a time series can overestimate the significance. We employed Durbin-Watson statistics to test for autocorrelation in the time series of solar radiation, sulfur dioxide, and atmospheric optical depth. The tests indicated rejecting the null hypothesis of no autocorrelation, suggesting that the time series are autocorrelated. We calculated the effective degrees of freedom, based on autocorrelation coefficient of residuals of detrended time series at lag one^55^, and despite the decreased degrees of freedom the correlations remain statistically significant at p = 0.025 or lower, supporting our results.

## Electronic supplementary material

Supplementary information
