# Supplementary material for: Drivers of solar radiation variability in the McMurdo Dry Valleys, Antarctica
Source: Sci Rep. 2018 Mar 22;8:5002. doi: 10.1038/s41598-018-23390-7 (PMC5864877; doi:10.1038/s41598-018-23390-7)
Supplement: Supplementary file 1 — Supplementary information [file 41598_2018_23390_MOESM1_ESM.pdf]

Drivers of solar radiation variability in the McMurdo Dry Valleys, Antarctica

Obryk, M. K., Fountain, A. G., Doran, P. T., Lyons, W. B., Eastman, R.

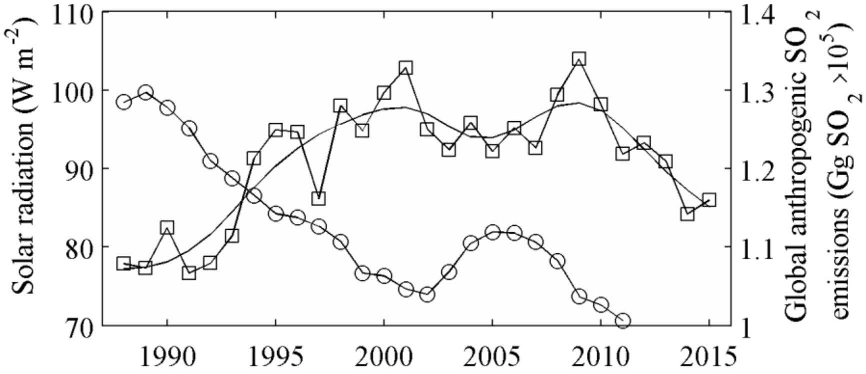

Figure S1. Solar radiation in McMurdo Dry Valleys (squares – left y-axis) and fitted moving average (solid curve). Global anthropogenic SO_2 emissions (circles – right y-axis). Correlation coefficient between the global SO_2 emissions and solar radiation fitted moving average: $r = -0.94$, $p < 0.001$, $n = 24$.

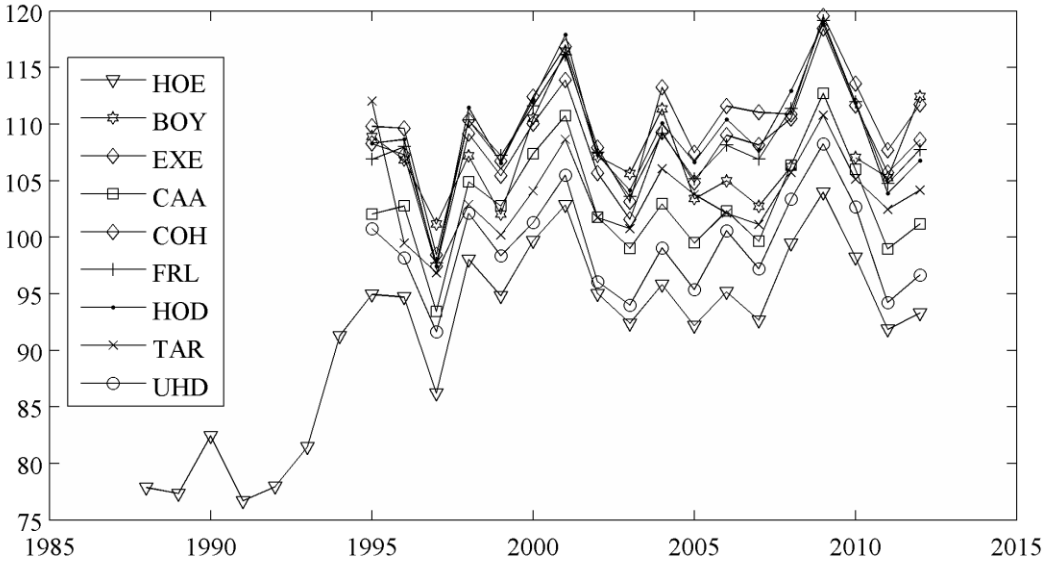

Figure S2. Annually averaged solar radiation for eight meteorological stations from Taylor Valley, Antarctica (see supplementary Table S1 for statistics).

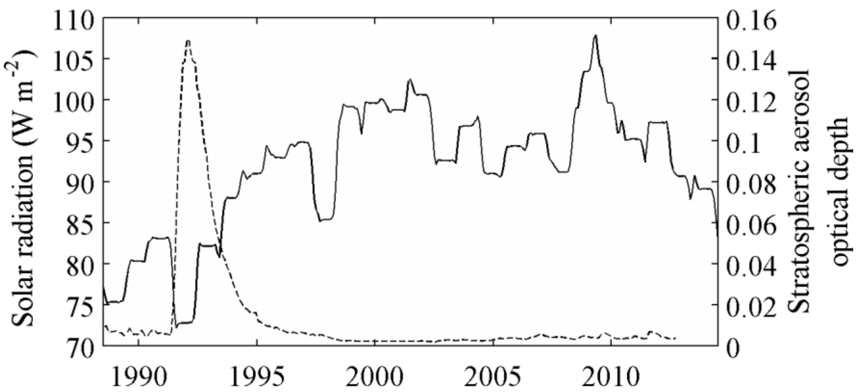

Figure S3. Solar radiation at Lake Hoare meteorological station expressed as a 13-term moving average (solid curve – left y-axis) and monthly stratospheric aerosol optical depth (dashed curve – right y-axis).

Table S1. Correlation coefficient between annually averaged solar radiation (as shown in supplementary Figure S2) from eight meteorological stations in Taylor Valley, Antarctica at $p < 0.05$.

	**HOE**	**BOY**	**EXE**	**CAA**	**COH**	**FRL**	**HOD**	**TAR**
**BOY**	0.67							
**EXE**	0.87	0.72						
**CAA**	0.99	0.68	0.88					
**COH**	0.92	0.68	0.98	0.93				
**FRL**	0.98	0.70	0.94	0.98	0.97			
**HOD**	0.97	0.66	0.93	0.97	0.95	0.98		
**TAR**	0.69	0.67	0.73	0.68	0.75	0.70	0.71	
**UHD**	0.95	0.57	0.87	0.95	0.93	0.94	0.96	0.73
